# Emergency response and preparedness among Polish human milk banks: a comparison of the COVID-19 pandemic and the 2022 Ukrainian refugee crisis

**DOI:** 10.3389/fnut.2024.1426080

**Published:** 2024-07-24

**Authors:** Małgorzata Gawrońska, Elena Sinkiewicz-Darol, Aleksandra Wesołowska

**Affiliations:** ^1^Faculty of Sociology, University of Warsaw, Warsaw, Poland; ^2^Human Milk Bank Foundation, Warsaw, Poland; ^3^Department of Physiology and Toxicology, Faculty of Biological Sciences, Kazimierz Wielki University, Bydgoszcz, Poland; ^4^Ludwik Rydygier’ Provincial Polyclinical Hospital in Torun, Torun, Poland; ^5^Laboratory of Human Milk and Lactation Research at Regional Human Milk Bank in Holy Family Hospital, Department of Medical Biology, Faculty of Health Sciences, Medical University of Warsaw, Warsaw, Poland

**Keywords:** human milk banking, emergency, COVID-19 pandemic, war in Ukraine, infant feeding

## Abstract

**Introduction:**

In recent years, Poland has faced two major emergencies: the COVID-19 pandemic, a global-scale public health emergency in 2020, and the outbreak of a full-scale war in Ukraine, which forced over 9 million Ukrainians–mostly women and children–to flee from their country through the Polish–Ukrainian border in 2022.

**Methods:**

In 2020 and 2022, we conducted two online questionnaires with human milk bank personnel to assess the impact of these emergencies on the human milk banking sector and its preparedness to face them. All 16 human milk bank entities operating in Poland were contacted and invited to participate in the study. For the first questionnaire, which was distributed in 2020, we obtained a 100% response rate. For the second questionnaire, the response rate was 88%, i.e., 14 out of 16 human milk banks completed the questionnaire. We compared these two emergencies in terms of the extent to which the potential of the Polish human milk bank network was exploited to support vulnerable infants who were not breastfed.

**Results and discussion:**

Our findings indicate that recommendations to provide donor human milk to infants separated from their mothers during the COVID-19 pandemic were never fully implemented. Meanwhile, during the refugee crisis, national legislation allowing equal access to public healthcare for Ukrainian citizens were rapidly implemented, enabling a more effective response by human milk banks to support vulnerable infants. However, no specific measures were introduced to support refugees outside the standard criteria for donor human milk provision. Our results highlight the limited response from the sector during emergencies and the underutilization of the potential of a nationwide network of professional human milk banks. Drawing on Polish experiences, we emphasize the importance of having procedures and legal regulations regarding human milk banking in place even in non-crisis settings, which would facilitate a rapid emergency response. We also emphasize the need to include the implementation of emergency procedures in building a strong and resilient human milk banking system.

## Introduction

In emergencies, children and women, especially women who are pregnant and lactating, are among the most at-risk social groups (1, 2). Feeding infants and young children in the absence of access to running water, reduced hygiene standards, and a lack of electricity is challenging. Breastfeeding, which requires no resources other than the presence of the mother, appears to be the only appropriate solution under these conditions (3). However, in practice, breastfeeding in crisis is often compromised and requires support (4–6). During a crisis, it is crucial to provide mothers with access to lactation counseling and to emphasize the importance of breastfeeding. This will make them aware of the risks of changing the baby’s diet and prevent the unwarranted use of formula. According to Infant and Young Children Feeding in Emergency (IYCF-E), it is recommended to implement solutions that can sustain breastfeeding and facilitate its return if it has been interrupted, such as relactation, wet nursing, and milk sharing. In this context, it is also worth considering that providing access to donor human milk (DHM) from a human milk bank (HMB) could serve as a “bridge to mother’s own milk (MOM)” (7, 8).

In Poland, the human milk banking sector has been developing rapidly for over 12 years. Initially, HMB entities were established by the non-governmental organization, the Human Milk Bank Foundation, with the support of the European Milk Bank Association (EMBA). Since 2018, some costs of the operation and opening of HMBs, including the purchase of equipment, have been financed from public funds under the governmental program “For Life” (9), and the procedure of tube feeding with DHM has been reimbursed by the Polish National Health Fund (10). Currently, there is a network of 16 units in Poland, located in almost all regions of the country, that operate under the auspices of the Human Milk Bank Foundation without a strict legislative framework (9, 11). All HMBs in Poland are hospital-based, non-profit laboratories that provide DHM to patients under the care of the highest level of the NICU. Although DHM is mentioned as a first alternative for vulnerable infants after their own mother’s milk in the Polish Standards of Perinatal Care, access to DHM is not granted by any hard-law legislation and is ultimately determined by whether a specific hospital has a signed contract with the regional HMB. This results in a wide disparity in access to DHM, even for preterm and critically sick newborns who are fed enterally by tubes and whose nutrition is covered by reimbursement from the Polish National Health Fund (10).

In recent years, Poland has faced two major emergencies: the COVID-19 pandemic, a global-scale public health emergency in 2020, and the outbreak of a full-scale war in Ukraine, which forced over 9 million Ukrainians–mostly women and children–to flee from their country through the Polish-Ukrainian border in 2022 (12), with over 1.5 million registered for temporary protection in Poland by February 2024 (13). Both of these emergencies affected the Polish healthcare system and posed a challenge to the human milk banking sector. The COVID-19 pandemic restrictions and initial concerns about the risk of SARS-CoV-2 virus transmission through human milk destabilized the human milk banking sector worldwide (14–17).

At the initial stage of the COVID-19 pandemic, in March 2020, Polish national consultants in the field of perinatal care issued their first recommendations on the treatment of mothers with confirmed or suspected infection and their infants, which introduced a number of measures that could heavily affect maternal and child wellbeing and future breastfeeding. Most importantly, the separation of mother and newborn after birth was recommended, with the possibility for the mother to express her milk to maintain lactation. The milk was recommended for disposal. It was feared that the SARS-CoV-2 virus could be transmitted not only through direct contact between mother and child but also through milk. In April 2020, the recommendations were updated, with a clear recommendation that infants separated from their mothers should be fed with DHM. In June 2020, the possibility of providing infants with expressed MOM was introduced, if only the mother’s clinical condition allowed it, and by September 2020, the separation policy was abolished and rooming-in and direct breastfeeding were made possible after obtaining the mother’s informed consent (16). These were all soft-law recommendations for clinical teams with no binding legal force.

The outbreak of a full-scale war in Ukraine in February 2022 resulted in a massive flow of refugees into the neighboring countries, which posed a challenge on many levels, including for the healthcare system, and required rapid legislative action (18–20). Many of the refugees fleeing the war were pregnant women and mothers of newborns, increasing vulnerable infant groups in need of DHM provision, as research shows that women with migrant backgrounds, and especially refugees, might face additional barriers to breastfeeding (2, 21–23). The Polish government very quickly introduced a number of measures, most importantly a bill allowing Ukrainian refugees to benefit from the Polish public healthcare system on the same basis as Polish citizens with a retrograde reimbursement system and including them in social protection schemes (24). Unlike the COVID-19 pandemic recommendations, these measures did not address the HMB sector directly and did not create new categories of potential DHM beneficiaries. However, as hard-law and legally binding instruments, these allowed Ukrainian refugee infants to access DHM in Poland on the same basis as Polish infants after meeting standard criteria (preterm born or sick infants).

In this paper, we examine the preparedness of the Polish HMB sector to face these emergencies, its response to them, and the use of the nationwide network potential of HMBs.

## Methods

To assess the impact of crises on the human milk banking sector and its readiness to face them, in September 2020 and September 2022, we conducted two online surveys with specialists working in Polish HMBs. The questionnaires were designed by experts from the Human Milk Bank Foundation, including professionals closely cooperating with two biggest Polish regional HMBs (AW and ESD) and a social scientist (MG). The questionnaires, consisting of open-ended and closed-ended questions, were distributed among Polish HMB staff utilizing LimeSurvey software. The usability and technical functionality of the survey, as well as the usability and clarity of the questions, were tested by one person from the Human Milk Bank Foundation team, other than the person who designed the survey, and one person working in a Polish HMB.

The surveys required the provision of information about the person responsible for filling out the questionnaire and the HMB entity they represented to ensure data reliability. Thus, the questionnaires were not anonymous, but all personal information and information about specific HMB entities were coded and only analyzed in an aggregated manner. The bioethical committee at the Warsaw Medical University had no objections to the conduct of the study (decision AKBE/240/2022).

The first questionnaire, focusing on the COVID-19 pandemic, was distributed in September 2020, and the second questionnaire focusing on the refugee crisis was distributed in October 2022. This means that both questionnaires were conducted in the period between 6 and 8 months after the outbreak of the emergency. Both emergencies had a chronic nature and were still not over when the questionnaires were distributed, but the culmination phase had passed. The questions addressed the impact of the emergencies on HMB operations, procedures, and DHM demand and supply, as well as cooperation with other entities in the region, changes in donor recruitment or human milk handling practices, and possible changes in the catalog of beneficiaries to address the needs of vulnerable groups in each emergency setting. While some questions were specific to a certain emergency, many were identical for both questionnaires to allow comparisons between the two events. The second questionnaire also included questions regarding emergency preparedness and assessment of the potential of HMB human milk banks’s potential during emergencies, which were not asked in the first questionnaire that focused on the COVID-19 pandemic. The response rate was 100% (*N* = 16) and 88% (*N* = 14) for the first and second questionnaires, respectively.

## Results

In this section, we describe the results obtained from two questionnaires distributed among Polish HMBs in 2020 and 2022. First, we compared the network response of Polish HMBs during both emergencies. Next, we described the impact of the COVID-19 pandemic and refugee crisis on the operation of HMB facilities in Poland. Finally, we focused on the assessment of the potential role of the HMB sector in emergencies in general and its preparedness to face them.

## A comparison of HMB responses to the COVID-19 pandemic and the refugee crisis

In both emergencies, we defined a group of the most vulnerable infants in need of DHM, as illustrated in Table 1. We also compared the two emergencies with regard to the type of regulations that granted access to DHM and their geographical scope. While the COVID-19 pandemic was a global event that affected the whole of Poland, the 2022 Ukrainian refugee crisis did not affect all regions of the country equally. First, in the initial stage of the crisis, most disruptions were the result of many people crossing borders in a short time. This mostly affected areas close to the border with Ukraine, as well as the largest cities easily accessed by trains. Second, the statistics show that the allocation of Ukrainian refugees across the country was not uniform, and different regions received very different numbers of temporary protection applications (13).

**Table 1:** Description of vulnerable groups of infants during emergencies.

	Description	Group beyond eligibility for DHM according to normally accepted criteria	Regulations granting access	Geographical scope of the emergency
The 2020 COVID-19 Pandemic	Infants separated from their mothers due to confirmed or suspected COVID-19 infection or hospitalized in neonatal wards with restricted access for mothers to provide expressed milk	Yes	Soft law (medical/ministry recommendations)	Nationwide
The 2022 Ukrainian Refugee Crisis	Infants who are Ukrainian citizens, who cannot be fed with their mother’s milk due to multiple reasons, born in Ukraine or Poland, whose mothers were refugees entering Polish territory due to the outbreak of full-scale war in 2022.	Not necessarily	Hard law (bill granting Ukrainian citizens equal access to the Polish healthcare system)	Regional concentration

When we compared the responses of the HMB sector to these two emergencies, we focused mostly on the extent to which the needs of specific vulnerable groups in terms of access to DHM were met. During the time period covered by our study, infants from these vulnerable groups received DHM in 6 (38%) and 10 (71%) HMBs, for the COVID-19 pandemic and the 2022 refugee crisis, respectively.

Demand for DHM is a valuable indicator for assessing the effectiveness of the HMB response in emergencies. Emergencies can expand the groups of potential beneficiaries of HMB services, in the case of the COVID-19 pandemic to include infants separated from their mothers, and in the case of the 2022 crisis to include refugee infants whose mothers may face additional barriers to breastfeeding. The lack of increased demand indicates that these new groups of infants in need may not have been recognized and ultimately had no access to DHM. In both emergencies–the COVID-19 pandemic and the 2022 refugee crisis–only a few Polish HMBs declared observing increased demand for DHM due to the emergency (Table 2).

**Table 2:** Increased demand for DHM as a result of the emergency.

	Yes % (N)	No % (N)	Hard to say % (N)
The COVID-19 Pandemic	19% (3)	56% (9)	25% (4)
The 2022 Refugee Crisis	21% (3)	57% (8)	21% (3)
Percentages in the table may not add up to exactly 100% due to rounding for clarity and readability purposes. However, the full numerical values have been used in the calculations and when considered, add up to 100%.			

Access to DHM supplies for vulnerable groups might have required modifications to the DHM eligibility criteria. This was especially important during the COVID-19 pandemic to ensure that all separated infants that could not receive their mothers’ milk–not only preterm and/or low birthweight–could benefit from HMB services. The majority of Polish HMBs did not modify the eligibility criteria for DHM due to the ongoing emergency, namely, they did not add the categories “separation after birth due to epidemiological restrictions” or “being a refugee” to the standard criteria such as preterm birth. During the COVID-19 pandemic, only five (31%) HMBs did so, but only in one case did a separated infant receive DHM. During the 2022 refugee crisis, only one (7%) HMB declared a revision of the criteria for donor milk provision in response to the crisis to include all hospitalized NICU infants (regardless of their refugee status), and one (7%) had already offered the possibility for any hospitalized infant, even beyond the standard criteria, to obtain DHM whenever there were medical indications. Out of 10 HMBs that provided DHM for Ukrainian refugees, 9 declared that those were typical situations, meeting DHM application criteria. Only one case was outside their scope, and DHM was provided based on the goodwill of the personnel, as no additional procedures were in place.

During the pandemic, another obstacle to HMB emergency response and providing vulnerable infants with access to DHM was the introduction of hospitals dedicated to the care of COVID-19 patients, including perinatal care. Selected hospitals in each voivodeship (region) of the country were designated as facilities to treat patients with COVID-19, including pregnant women who were sick or infected with SARS-CoV-2. Initially, there were 21 such hospitals. Prior to the pandemic, only 4 out of 16 Polish HMBs had formal contracts signed with the hospitals that later admitted pregnant and postpartum women suffering from COVID-19. Most of these facilities designated to treat patients with COVID-19 did not have either HMBs in their structure or any form of contract with regional HMBs. During the pandemic, in the period covered by our survey, no HMB established such formal cooperation, and only one established informal cooperation with such a facility. Among the obstacles to establishing such cooperation, the main one was the lack of interest in cooperation from the hospitals dedicated to the care of COVID-19 patients (*N* = 6). Other reasons were differences in communication between clinical teams and administrations of the hospital where the bank and the COVID-19 hospital were located (*N* = 3), insufficient volume of milk collected in the HMB (*N* = 2), difficulties in the logistics of milk transportation (*N* = 1), and confusion regarding which hospital in the region was dedicated to caring for COVID-19 patients (*N* = 1). The lack of a contract between the COVID-19-dedicated hospitals and regional HMBs made it impossible for infants hospitalized in those facilities to obtain DHM from HMBs.

## Impact of the COVID-19 pandemic and refugee crisis on HMB operations in Poland

Our study shows that the COVID-19 pandemic had a greater impact on HMB’s operation in Poland than the refugee crisis. During the refugee crisis, almost all Polish HMBs declared no impact on their operation (*N* = 13, 93%), while during COVID-19, this was the case in 10 (63%) HMBs. It should be noted that both emergencies were of a different nature, and while the pandemic often led to restrictions on HMB operations, the refugee crisis was a situation in which hospitals, including those with HMBs, faced various difficulties related to the admission of foreign patients, and staff were involved in additional volunteer activities.

During the pandemic, the majority (*N* = 12) of HMBs remained open throughout the entire period covered by our study, with only a few declaring temporary closures for a short period of time, as shown in Table 3.

**Table 3:** Closure of the human milk bank entity during the COVID-19 pandemic.

	% (N)
HMB permanently open	75% (12)
HMB temporarily closed	25% (4)
Closed for a few days	6% (1)
Closed for a few weeks	13% (2)
Closed for over a month	6% (1)

Other changes in the HMB operation during the pandemic included shortening the opening hours or limiting the number of personnel (*N* = 3) and temporary suspension of donor recruitment and/or acceptance of milk (*N*=1). The most important factor impacting HMB operation during the pandemic was restricted access to the hospital, making it difficult for donors to visit the HMB to donate or get tested (*N* = 8), followed by pandemic-related shortages in HMB’s personnel. These included obligatory quarantine of personnel after contact with a person with a confirmed COVID-19 infection, sick leave, or leave for childcare purposes, as the educational and childcare facilities were closed and operated in an online mode.

All reasons for restricting the operation of HMBs during the pandemic are listed in Table 4.

**Table 4:** Reasons for restricting HMBs operations during the COVID-19 pandemic.

	% (N)
Administrative decisions by hospital management	13% (2)
COVID-19-related shortages of HMB personnel	31% (5)
Restrictions on access to the hospital affecting donor testing or visits to provide milk	50% (8)
Difficulties with logistics in milk transportation	25% (4)
Decrease in the number of potential and already recruited donors	25% (4)

Over half of Polish HMBs (*N* = 10, 63%) observed a decrease in the number of donors during the COVID-19 pandemic. It was mostly because fewer potential donors contacted the HMB–a factor observed in all entities where the number of donors decreased–and due to resignations from further donations by already recruited donors (*N* = 4, 25%). Despite this, the majority (*N* = 12, 75%) of the entities did not observe shortages in DHM stock.

Hospitals organized the transportation of milk from donors to eight Polish HMBs. During the pandemic, two milk banks introduced a solution to transport milk from donors to the HMB by selecting personnel who used their private vehicles during work hours. In one HMB, such a possibility was introduced as a result of a bottom-up initiative of the personnel and was performed after work hours.

The pandemic also impacted the recruitment process of donors. Only one HMB declared that no changes in recruitment were introduced. Among the most commonly introduced changes was an additional epidemiological interview aimed at establishing whether the potential donor had infection symptoms, had contact with individuals exhibiting such symptoms, was ill or in quarantine, or had recently traveled to areas with a high risk of COVID-19 exposure (*N* = 15). Other changes included additional instructions on sanitary rules regarding human milk expression and storage (*N* = 10) and conducting the initial stage of recruitment online (*N* = 7). Only two HMBs declared testing donors for COVID-19, and in four HMBs, potential donors were limited to those hospitalized in obstetric wards.

The majority of HMBs also declared implementing changes in cooperation with donors recruited before the pandemic (*N* = 12). In total, 11 HMBs introduced additional instructions on sanitary rules regarding human milk expression and storage for previously recruited donors, and 10 conducted additional epidemiological interviews with them. Over half of HMBs also declared implementing changes in milk handling (*N* = 7). In four HMBs, changes were introduced in the milk sample storage conditions–for example, samples ‘quarantine’ before pooling and pasteurization in order to establish if the donor would develop symptoms of infection or changes in milk transportation rules (*N* = 3). One of the HMBs introduced rules regarding the DHM’s sample labeling.

None of the HMBs declared that the refugee crisis had any impact on donor recruitment procedures. However, one HMB reported that some donors had contacted the entity with a direct wish to donate milk for refugee children or war victims. During the refugee crisis, some HMB staff declared that there were hospital-level changes in the organization of work that affected the work of HMB (*N* = 3). These included providing interpreter or electronic translator services, translating medical documentation into the Ukrainian language, providing informational materials for patients in different languages, or hiring additional Ukrainian-speaking personnel (including lactation consultants). In two HMBs, these changes were not the effect of official decisions but were based on a bottom-up mobilization of Ukrainian-speaking personnel. In four cases, the person completing the questionnaire was not aware of any changes in the organization of the work.

Half (*N* = 7) of the HMB participating in the study engaged in additional activities aimed at supporting refugees, mostly by participating in fundraisings and non-cash goods collections (*N* = 2), undertaking breastfeeding promotion actions (*N* = 2), providing access to lactation counseling, and providing breast pumps and supportive accessories for refugee mothers (*N* = 2). The refugee crisis did not affect either positively or negatively the number of donors or the profile of donors in any Polish HMB.

## Assessment of the preparedness of the HMB sector for emergencies and its potential role

The results indicate the low level of preparedness of the HMB sector in Poland for emergencies, especially regarding the application of Infant and Young Child Feeding in Emergencies (IYCF-E) procedures, as illustrated in Table 5. In four (29%) HMBs, the person responsible for completing the questionnaire was not aware of any IYCF-E procedures applied in the neonatal unit/hospital, where the HMB was located. Six HMBs (43%) declared that no such procedures were applied. None of the HMBs reported the application of procedures based on international guidelines, such as the Operational Guidelines of Infant Feeding in Emergency (OG-IFE). In one case (7%), procedures based on local/regional guidelines (such as recommendations from regional consultants in public health) were applied, and in two cases (14%), internal procedures at the hospital or HMB level were in place. It is important to note that three (21%) HMBs declared the application of procedures based on the national guidelines; however, at the time of conducting the study and writing this paper, no such guidelines existed in Poland. This might suggest a low level of awareness among HMB personnel regarding the topic and indicate the need for further education of HMB professionals in this field.

**Table 5:** Application of procedures for the feeding of infants and young children in emergencies.

Lack of any procedures applied	43% (6)
Lack of knowledge about any procedures being applied	29% (4)
Application of procedures based on international guidelines	0
Application of procedures based on national guidelines (non-existent)	21% (3)
Application of procedures based on local/regional guidelines	7% (1)
Application of internal (hospital or HMB level) procedures	14% (2)

The results indicate that there are currently no legally binding or well-established rules regarding the human milk banking sector in emergencies in Poland, and in the majority of Polish HMBs, there are probably no procedures in place for organizing the work and decision-making process in the event of an emergency. However, the HMB personnel relatively rarely highlighted the lack of dedicated emergency procedures at the national (*N* = 3, 21%) or hospital (*N* = 3, 21%) level as the main obstacle to fulfilling the sector’s potential during the 2022 refugee crisis. More often, it was noted that already existing national procedures about that situation were not clear (*N* = 6, 43%). Most importantly, the language barrier between HMB personnel and refugees was most often cited as an obstacle (*N* = 7, 50%).

During the 2022 refugee crisis, HMB personnel described the role of the sector as broader than just providing DHM to refugee infants in need. Only six respondents (43%) saw the role of the human milk banks during this emergency as solely providing DHM. More often, the role of HMB was seen in supporting refugee mothers facing difficulties with breastfeeding (*N* = 11, 79%) and the general promotion of breastfeeding among Ukrainian mothers (*N* = 11, 79%). Other fields where the potential of the HMB sector was identified included supporting the integration of refugees into new communities (*N* = 8, 57%) and cooperation with hospitals in providing psychological support to refugee mothers (*N* = 6, 43%). Among these fields, HMB personnel assessed the utilization of the sector’s potential best in the areas of general promotion of breastfeeding among Ukrainian mothers (*N* = 10, 71%), support for refugee mothers facing difficulties with breastfeeding (*N* = 8, 57%), and provision of DHM to refugee infants in need (*N* = 7, 50%). The overall assessment of the utilization of the HMB sector’s potential during the crisis was ambiguous, with the majority of the respondents choosing the ‘hard to say’ option (*N* = 6, 43%) and an equal number of respondents assessing it as high (*N* = 4, 29%) and low (*N* = 4, 29%). At the same time, it was also inconclusive how HMB professionals in our sample perceived the role of the HMB sector during the refugee crisis in comparison with other healthcare sectors. The majority chose the ‘hard to say’ option (*N* = 9, 64%), while three (21%) assessed it as high and two (14%) as low.

## Discussion

This study compares Polish HMBs’ responses to two unexpected situations—one related to the COVID-19 pandemic and the other to the refugee flow to Poland resulting from the ongoing full-scale war in Ukraine. Although some characteristics of these events were different, both situations were considered crises as they exceeded the capacity for an effective response. We draw on the results of two questionnaires that were conducted during the period between 6 and 8 months after the outbreak of the emergencies. Both emergencies were chronic and ongoing when the questionnaires were distributed, although the culmination phase had already passed. We observed that the COVID-19 pandemic had a greater impact on HMB operations (37% of all HMBs declared such) than the refugee crisis resulting from the full-scale war in Ukraine (7% of participating entities declared such), which also might have impacted the sector’s response. We treat these two events as a lens through which the fragility of the national human milk banking system was exposed, with a lack of binding legal regulations, sufficient financial streams, the implementation of international guidelines and effective nationwide policies. In fact, our research revealed the Polish human milk banking sector’s relatively low preparedness for emergencies. Six (43%) entities declared a lack of any procedure in place in case of an emergency. None of the Polish HMBs implemented procedures based on international guidelines, such as the OG-IFE. This is understandable given that the IYCF-E working expert team was only agreed upon by the Polish Ministry of Health in September 2022 to develop a strategy for infant and young child feeding during a crisis.

Similar to other countries, Poland quickly introduced many restrictions in the healthcare system, resulting from the outbreak of the COVID-19 pandemic. Many of these affected perinatal care and breastfeeding. As the COVID-19 pandemic evolved, HMBs worldwide continued to provide donor milk to infants without access to their mother’s own milk. Ensuring the safety of pasteurized milk was one of the priorities (14).

Despite initial concerns, studies have quickly shown no risk of vertical transmission of SARS-CoV-2 by human milk (25), as well as the effectiveness of proper hygiene measures in avoiding infection transmission from mother to child (26, 27), and proved that holder pasteurization inactivates the SARS-CoV-2 virus (28). During the pandemic, the HMB sector proved to be safe, with a high level of self-regulation, operating on well-established, evidence-based procedures, and being effective in introducing necessary changes to maintain safety in the initial stage of the pandemic when limited evidence on virus transmission was available (17, 29). In line with this, almost all Polish HMBs introduced extraordinary measures during the pandemic, such as additional epidemiological interviews and instructions on sanitary rules regarding human milk expression and storage. Some standard procedures related to the recruitment of donors and storage of DHM samples were changed following the recommendations of the EMBA (30).

Many HMBs worldwide initially struggled to respond to the COVID-19 pandemic, especially in its early stages, with limited knowledge and evidence, a lack of agreed-upon safety guidelines regarding DHM recipients, and inadequate capacity to ensure the ability to respond in times of crisis (14–17, 31, 32). This is due to a lack of clear international guidelines and legal frameworks regarding the role of HMBs in times of global crisis. During the pandemic the number of babies that could be fed with DHM because of being deprived of MOM, either due to separation policies or their mothers’ condition, increased (33, 34). At the same time, the recruitment of milk donors and the overall availability of mother’s milk were negatively affected, making it difficult to increase the stock of DHM and meet the target of potentially increasing the number of DHM recipients, by including those babies. Our results show that the increased demand was not even noticed by the Polish HMB sector (see Table 2) because only a few Polish HMBs modified the DHM eligibility criteria. This is confirmed by the annual data collected by the Human Milk Bank Foundation on the volume of DHM donated in Polish HMBs. Comparing 2019 and 2020, the volume was almost the same, with 4,651 L of DHM collected in 2019 vs. 4,605 L of DHM collected in 2020 (data from the Human Milk Bank Foundation, unpublished). Comparing 2021 and 2022, the volume of milk collected decreased in the year of emergency, with 5,241 L of DHM collected in 2021 vs. 4,388 L collected in 2022. The increased demand for DHM during the pandemic and refugee crisis may be an indication of the effectiveness of utilizing the nationwide network of HMBs in Poland. Both emergencies expanded the groups of potential beneficiaries of HMB services to include refugee infants whose mothers may face additional barriers to breastfeeding, or infants separated from their mothers with confirmed or suspected COVID-19 infection. In both situations, these infants may meet the standard criteria for DHM application (preterm born or sick), but there may also be other emergency situations requiring modifications to the standard list of beneficiaries.

Our research proves that during emergencies, it is not the safe operation of the HMB sector that is endangered, but rather its ability to respond to the crisis by effectively supporting its victims. Despite difficulties faced during emergencies, especially the COVID-19 pandemic, which affected HMB operation and donor recruitment, Polish HMB succeeded in maintaining safe operation; only a few HMBs were closed temporarily for a short time during the initial stage of the pandemic (see Table 3), and the majority of HMBs did not observe shortages in DHM supply and managed to sustain the collection of DHM at the same level as before the pandemic. However, this data clearly shows that the response of the sector during emergencies and the provision of DHM for new groups of beneficiaries were limited. Our study showed that in emergencies, HMBs in Poland were not able to establish effective cooperation with other hospitals, including signing contracts and starting to deliver milk to hospitals where infants born to mothers with confirmed or suspected COVID-19 infection were hospitalized. Although the COVID-19 pandemic restrictions in Poland were still very strict until 2021, the recommendation of the National Consultant for Neonatology related to feeding with DHM for those children who do not have access to their mother’s own milk due to COVID-19 emerged quite rapidly. During the period covered by our study, in only one HMB (out of the five that implemented such changes), did the expansion of the criteria for DHM supply lead to its provision to an infant separated from the mother due to COVID-19. Out of the six entities where DHM was provided to hospitalized infants whose mothers were not able to provide their expressed milk due to epidemiological restrictions, in four instances, these infants should have been considered premature or sick as the criteria were not changed.

Refugee infants benefited from DHM in 10 entities participating in the study; however, in nine HMBs, standard criteria for DHM supplementation were applied (preterm delivery), and one was an exceptional case that did not meet the standard eligibility criteria and DHM was provided due to the personnel’s goodwill, as no procedures were in place. During the 2022 refugee crisis, no official guidelines regarding the provision of DHM to refugee infants were issued, despite existing research proving that refugee mothers might face additional barriers to breastfeeding their children (2, 21, 23) and that in emergencies, DHM is a recognized option for infant feeding (35).

However, the role of the HMB sector during emergencies, as our research shows, can be broader than just providing DHM. In emergencies, proper lactation support is crucial for maintaining breastfeeding, including providing high-quality consultations and care for refugee mothers (2, 21, 23). In the case of both Iraqi and Syrian refugees to Turkey, it has been shown that lactation care, information on the benefits of breastfeeding, and support for mothers’ skills can contribute to an increase in breastfeeding rates, and the important role of HMB in educating refugee women has been highlighted (36, 37). Furthermore, in Poland, during the 2022 refugee crisis, 10 out of 16 hospitals with HMBs joined a humanitarian project aimed at supporting mothers in a deep lactation crisis caused by war trauma or other emergencies, implemented by the Human Milk Bank Foundation in cooperation with the UNICEF Office for Refugee Response (38). Our results show that the HMBs were engaged in multiple activities aimed at refugee support, overall breastfeeding support, and promotion of breastfeeding among refugee mothers, indicating an underutilized potential of the sector that lacked a coordinated response.

The role of the HMB sector during crises has also recently been highlighted in Israel, which is undergoing military operations. In accordance with the guidelines and procedures adopted when establishing a national HMB in Israel, DHM was initially intended for the feeding of preterm and high-risk infants. After the outbreak of hostilities, regulations were introduced at the national level by the Ministry of Health. In line with the guidelines announced by the Israeli Ministry of Health, the HMB has expanded its operations to be able to provide DHM to infants up to 6 months of age in cases where their mothers have been killed, missing, injured, kidnapped, or called for reserve duty. Additionally, it was indicated that mothers’ milk should be given to children in those situations between 6 months and 1 year of age, in case of allergy or sensitivity to formula or for children being sick or injured, if it is necessary for their recovery (39, 40). In order to meet the regulations established by the Ministry of Health, the Israeli HMB indicated the need to work three times more intensively, especially in recruiting new donors and establishing cooperation with new medical units, which unfortunately was not observed in Poland during the two emergencies we investigated.

Our data, when compared with the experiences of other countries, demonstrate the need for the presence of legal solutions and firm procedures in place at the national level. In the case of the COVID-19 pandemic, infants who were isolated from their mothers and hospitalized for long periods without the possibility of visits/supply of MOM to the ward were fed with DHM based on the recommendations of national consultants; these recommendations were never included in the current hard-law regulations in Poland, including reimbursement schemes. As our data demonstrate, these soft-law recommendations were never fully implemented in practice. This could be possibly due to a lack of legal regulations in the HMB sector in Poland, including the implementation of effective procedures for emergencies. Thus, it was impossible to account for these services unless there was a standard rationale for the supply of DMH from a bank. The lack of legal regulations at the national level has also been identified in other parts of the world during the pandemic as an obstacle to the operation of HMBs in accordance with professional recommendations (15, 17, 32, 41).

In the case of the 2022 refugee crisis, hard-law regulations were very quickly put in place at the national level to allow the supply of DHM to Ukrainian infants on the same basis as Polish children in order to facilitate refugees’ access to healthcare services (18–20, 24). However, no specific guidelines for the human milk banking sector have been issued, especially none that would allow for the provision of DHM to refugee infants that do not meet the standard eligibility criteria (preterm or sick). The experiences of other countries prove that only the implementation of systemic, nationwide solutions specifically addressing the human milk banking sector will allow effective action in the acute phase of the crisis. Such effective action should be based on solutions from preparedness: an HMB in the Philippines has a procedure in place to collect a 10% reserve from each donation for a crisis (42, 43). This is a direct result of the country’s full implementation of IYCF-E guidelines in the field of DHM provision in emergencies and the acknowledgment of the important role of DHM as the best feeding alternative for infants who are not breastfed, alongside relactation and wet nursing (44). It has become evident that implementing global standard solutions for the human milk banking sector is crucial for better protecting breastfeeding in emergencies. Unfortunately, the human milk banking sector is still not treated as a priority in emergency preparedness planning and emergency response, despite its possible role not only in DHM provision but also in professional breastfeeding support and guidance for mothers affected by the crisis. Our results show that even HMB professionals often underestimate the role they and their expertise could have during emergencies. This attitude may also be the result of a high dependence on formulas during emergencies (45, 46). As recent experiences with US formula shortages showed, breastfeeding (and strengthening human milk banking, as well as other practices relying on lactating women such as milk sharing and wet nursing) is more resilient to such perturbations. The availability of formula in the market is sensitive to supply chain disruptions, especially during crises (35, 47), and should be considered a last resort of choice.

Based on our experiences from the Polish study, we emphasize the significance of having procedures and legal regulations in place for human milk banking, even in non-crisis situations. These would enable quick response in emergencies and help to strengthen the DHM provision system by implementing emergency procedures. Our research had some limitations, mainly related to the sample size. Although we obtained a high response rate, there are only 16 HMBs in Poland, and our sample size did not allow for statistical analysis. Further research, in an international context, is urgently needed to learn about the experiences of the HMB sector in various emergencies, its preparedness for possible future emergencies, and to share past mistakes and good practices.

## Data availability statement

The raw data supporting the conclusions of this article will be made available by the authors, without undue reservation.

## Ethics statement

The studies involving humans were approved by The Bioethical Committee at the Warsaw Medical University which expressed no objections to its conduct (decision AKBE/240/2022). The studies were conducted in accordance with the local legislation and institutional requirements. The participants provided their written informed consent to participate in this study.

## Author contributions

MG: Conceptualization, Data curation, Formal analysis, Investigation, Methodology, Visualization, Writing – original draft. ES-D: Data curation, Formal analysis, Validation, Writing – review & editing. AW: Conceptualization, Methodology, Project administration, Resources, Supervision, Writing – review & editing, Writing – original draft.

## References

[1] UNFPA. *Protecting Women in Emergency Situations. U N Popul Fund*. (2024). Available at: https://www.unfpa.org/resources/protecting-women-emergency-situations#:~:text=HEIGHTENED%20RISK%2C%20GREATER%20NEED&text=Women%20and%20children%20account%20for,is%20likely%20to%20be%20pregnant.

[2] Palmquist AEL Gribble KD. Gender, displacement, and infant and young child feeding in emergencies In: Riley NE Brunson J, editors. International handbook on gender and demographic processes. International handbooks of population. Dordrecht: Springer Netherlands (2018). 341–55.

[3] Tomori C. Global lessons for strengthening breastfeeding as a key pillar of food security. Front Public Health. (2023) 11:1256390. doi: 10.3389/fpubh.2023.1256390, PMID: 37674689 PMC10477442

[4] DeYoung SE Chase J Branco MP Park B. The effect of mass evacuation on infant feeding: the case of the 2016 Fort McMurray wildfire. Matern Child Health J. (2018) 22:1826–33. doi: 10.1007/s10995-018-2585-z, PMID: 30054788

[5] Giusti A Marchetti F Zambri F Pro E Brillo E Colaceci S. Breastfeeding and humanitarian emergencies: the experiences of pregnant and lactating women during the earthquake in Abruzzo, Italy. Int Breastfeed J. (2022) 17:45. doi: 10.1186/s13006-022-00483-8, PMID: 35706034 PMC9199337

[6] Iellamo A Monaghan E Moghany SAL Latham J Nassereddin N. Breastfeeding knowledge of mothers in protracted crises: the Gaza strip example. BMC Public Health. (2021) 21:742. doi: 10.1186/s12889-021-10748-2, PMID: 33865341 PMC8052738

[7] IFE Core Group. *Operational Guidance on Infant Feeding in Emergencies (OG-IFE) version 3.0*. (2017). Available at: https://www.ennonline.net/operationalguidance-v3-2017.

[8] Brown A Shenker N. Receiving screened donor human milk for their infant supports parental wellbeing: a mixed-methods study. BMC Pregnancy Childbirth. (2022) 22:455. doi: 10.1186/s12884-022-04789-7, PMID: 35641919 PMC9154035

[9] Gawrońska M Wesołowska A. Umocowania prawno-instytucjonalne funkcjonowania banków mleka w Polsce In: Wilińska M., editor. Laktacja tom 2. Wiedza kliniczna i farmakoterapia. Warszawa: PZWL Wydawnictwo Lekarskie (2024)

[10] Wesołowska A Bernatowicz-Łojko U Sinkiewicz-Darol E Pawlus B Golicki D. Implementation of the reimbursement cost of human-Milk-based neonatal therapy in polish health care service: practical and ethical background. J Hum Lact. (2020) 36:426–35. doi: 10.1177/0890334420909815, PMID: 32491973

[11] Klotz D Wesołowska A Bertino E Moro GE Picaud J Gayà A. The legislative framework of donor human milk and human milk banking in Europe. Matern Child Nutr. (2022) 18:e13310. doi: 10.1111/mcn.13310, PMID: 34936203 PMC8932705

[12] Straż Graniczna. *Informacja statystyczna za 2022 r. Komenda Główna Straży Granicznej*. (2023). Available at: https://www.strazgraniczna.pl/pl/granica/statystyki-sg/2206,Statystyki-SG.html (Accessed April 22, 2024).

[13] UNHCR. *Refugees from Ukraine registered in Poland, by district (powiat)*. (2024). Available at: https://data.unhcr.org/en/dataviz/226?sv=54&geo=10781 (Accessed April 22, 2024).

[14] Shenker N Staff M Vickers A Aprigio J Tiwari S Nangia S. Maintaining human milk bank services throughout the COVID-19 pandemic: a global response. Matern Child Nutr. (2021) 17:e13131. doi: 10.1111/mcn.13131, PMID: 33403779 PMC7883204

[15] Olonan-Jusi E Zambrano PG Duong VH Anh NTT Aye NSS Chua MC. Human milk banks in the response to COVID-19: a statement of the regional human milk bank network for Southeast Asia and beyond. Int Breastfeed J. (2021) 16:29. doi: 10.1186/s13006-021-00376-2, PMID: 33781285 PMC8006108

[16] Sinkiewicz-Darol E Bernatowicz-Łojko U. Operation of the first regional Milk Bank in Poland during a SARS-CoV-2 (COVID-19) pandemic. J Hum Lact. (2020) 36:626–7. doi: 10.1177/0890334420957971, PMID: 32960122

[17] Cohen M Cassidy T. The impact of the Covid-19 pandemic on north American milk banks. Matern Child Nutr. (2021) 17:e13234. doi: 10.1111/mcn.13234, PMID: 34190391 PMC8420577

[18] Lach DE. Prawo uchodźców wojennych z Ukrainy do świadczeń opieki zdrowotnej w Polsce. Pr Zabezp Społeczne. (2023) 2023:54–61. doi: 10.33226/0032-6186.2023.1.7

[19] Firlit-Fesnak G Jaroszewska E Łotocki Ł Łukaszewska-Bezulska J Ołdak M Zawadzki P. *Inwazja Rosji na Ukrainę. Społeczeństwo i polityka wobec kryzysu uchodźczego w pierwszym miesiącu wojny. Raport roboczy - Working Paper Katedry Polityki Społecznej*. Warszawa: Wydział Nauk Politycznych i Studiów Międzynarodowych, Uniwersytet Warszawski. (2022). Available at: https://wnpism.uw.edu.pl/wp-content/uploads/2022/04/Kryzys-uchodzczy-2022-raport-KPS.pdf (Accessed April 26, 2024).

[20] Kolińska K Paprocka-Lipińska A Koliński M. The Ukrainian refugee crisis: its ethical aspects and challenges for the polish healthcare system – a descriptive review. Eur J Transl Clin Med. (2023) 6:79–86. doi: 10.31373/ejtcm/162233

[21] Hirani SAA. Barriers affecting breastfeeding practices of refugee mothers: a critical ethnography in Saskatchewan, Canada. Int J Environ Res Public Health. (2024) 21:398. doi: 10.3390/ijerph21040398, PMID: 38673311 PMC11050554

[22] Izumi C Trigg J Stephens JH. A systematic review of migrant women’s experiences of successful exclusive breastfeeding in high-income countries. Matern Child Nutr. (2024) 20:e13556. doi: 10.1111/mcn.13556, PMID: 37584632 PMC10750009

[23] Halasa S Safadi R Al-Maharma D Ahmad M Saleh M Nabolsi M. *Rates, facilitators, and barriers to exclusive breastfeeding among Syrian refugee mothers: a cross-sectional survey*. (2022).

[24] Howard S. Poland’s buckling healthcare system nevertheless welcomes Ukraine refugees with open arms. BMJ. (2022) 377:o844. doi: 10.1136/bmj.o844, PMID: 35365477

[25] Jeganathan K Paul AB. Vertical transmission of SARS-CoV-2: a systematic review. Obstet Med. (2022) 15:91–8. doi: 10.1177/1753495X211038157, PMID: 35795545 PMC9247633

[26] Yang N Che S Zhang J Wang X Tang Y Wang J. Breastfeeding of infants born to mothers with COVID-19: a rapid review. Ann Transl Med. (2020) 8:618–8. doi: 10.21037/atm-20-3299, PMID: 32566555 PMC7290644

[27] Al-kuraishy HM Al-Gareeb AI Atanu FO MA EL-Z Diab HM Ahmed AS. Maternal transmission of SARS-CoV-2: safety of breastfeeding in infants born to infected mothers. Front Pediatr. (2021) 9:738263. doi: 10.3389/fped.2021.738263, PMID: 34956971 PMC8696119

[28] Walker KF O’Donoghue K Grace N Dorling J Comeau JL Li W. Maternal transmission of SARS-COV-2 to the neonate, and possible routes for such transmission: a systematic review and critical analysis. BJOG Int J Obstet Gynaecol. (2020) 127:1324–36. doi: 10.1111/1471-0528.16362, PMID: 32531146 PMC7323034

[29] Picaud J-C Buffin R Rigourd V Boscher C Lamireau D Dumoulin D. It’s time to change the recommendations on COVID-19 and human milk donations. Acta Paediatr. (2021) 110:1405–6. doi: 10.1111/APA.15782, PMID: 33527456 PMC8014273

[30] European Milk Banking Association. *COVID-19: EMBA position statement*. European Milk Banking Association (2020).

[31] Shenker N Aprigio J Arslanoglu S Aye NSS Bærug A Bar Yam N. Maintaining safety and service provision in human milk banking: a call to action in response to the COVID-19 pandemic. Lancet Child Adolesc Health. (2020) 4:484–5. doi: 10.1016/S2352-4642(20)30134-6, PMID: 32573440 PMC7202855

[32] Shenker N Hughes J Barnett D Weaver G. Response of UK milk banks to ensure the safety and supply of donor human milk in the COVID-19 pandemic and beyond. Infant. (2020) 16:118–21.

[33] Bhasin M Nangia S Kumar G Parihar A Goel S. Sequential interventions to maintain the safety and service provisions of human milk banking in India: keeping up with the call to action in response to the COVID-19 pandemic. Int Breastfeed J. (2022) 17:85. doi: 10.1186/s13006-022-00525-1, PMID: 36517901 PMC9748401

[34] Chanda BM Chen X-Q. Breastfeeding during the COVID-19 pandemic. Front Pediatr. (2023) 11:1120763. doi: 10.3389/fped.2023.1120763, PMID: 37342530 PMC10277472

[35] Smith JP Iellamo A. Wet nursing and donor human milk sharing in emergencies and disasters: a review. Breastfeed Rev. (2020) 28:7–23.

[36] Varer Akpinar C Mandiracioglu A Ozvurmaz S Adana F Koc N Kurt F. Attitudes towards human milk banking among native Turkish and refugee women residing in a rural region of Turkey: a mixed-methods approach. Int Breastfeed J. (2022) 17:74. doi: 10.1186/s13006-022-00516-2, PMID: 36303181 PMC9615202

[37] Yalçın SS Erat Nergiz M Yalçın S. Evaluation of breastfeeding and infant feeding attitudes among Syrian refugees in Turkey: observations of Syrian healthcare workers. Int Breastfeed J. (2023) 18:38. doi: 10.1186/s13006-023-00579-9, PMID: 37559070 PMC10413606

[38] Human Milk Bank Foundation. *Infant and young children feeding in emergency*. (2024). Available at: https://bankmleka.pl/en/feeding-children-in-emergency/ (Accessed April 28, 2024).

[39] Ghert-Zand R. *Health ministry issues directives for national breast milk bank during war*. Times Isr (2023). Available at: https://www.timesofisrael.com/health-ministry-issues-directives-for-national-breast-milk-bank-during-war/ (Accessed April 28, 2024).

[40] Magen David Adom in Israel. *MDA’s National Milk Bank saving lives in war*. Magen David Adom Isr (2023). Available at: https://www.mdais.org/en/news/311023-1 (Accessed April 28, 2024]

[41] Tikvina S Zilli E Golin R Ramina P Tikvina S Cecchinato P. Human milk banks during covid-19 pandemic. Popul Med. (2023) 5:613. doi: 10.18332/popmed/164613

[42] Milanes V. *How a human milk banks works. Human milk banks ensure that infants in emergency situations receive breast milk*. Unicef Philipp (2019). https://www.unicef.org/philippines/stories/how-human-milk-bank-works (Accessed April 28, 2024).

[43] Human Milk Bank Foundation. *Human milk bank in emergency – Donor milk as a bridge to mothers own milk*. (2023). https://bankmleka.pl/en/2023/05/17/human-milk-bank-in-emergency-donor-milk-as-a-bridge-to-mothers-own-milk-22-june-2023/ (Accessed April 28, 2024).

[44] Romelei Camiling-Alfonso AMFT Donna Isabel S Capili KA Reyes V Silvestre MA. Contributing to the infant and young child feeding in emergencies (IYCF-E) response in the Philippines: a local NGO perspective (2015) 50:96.

[45] Iellamo A Wong CM Bilukha O Smith JP Ververs M Gribble K. “I could not find the strength to resist the pressure of the medical staff, to refuse to give commercial milk formula”: a qualitative study on effects of the war on Ukrainian women’s infant feeding. Front Nutr. (2024) 11:940. doi: 10.3389/fnut.2024.1225940, PMID: 38826579 PMC11140133

[46] Gribble K Peterson M Brown D. Emergency preparedness for infant and young child feeding in emergencies (IYCF-E): an Australian audit of emergency plans and guidance. BMC Public Health. (2019) 19:1278. doi: 10.1186/s12889-019-7528-0, PMID: 31610779 PMC6792236

[47] Jackson F Obeng C. Perceptions of human Milk banks as a response to the US infant formula shortage: a mixed methods study of US mothers. Women. (2022) 2:218–30. doi: 10.3390/women2030022

